# Large-bore aspiration thrombectomy for the treatment of pulmonary embolism in octogenarians

**DOI:** 10.1186/s42155-025-00517-2

**Published:** 2025-01-22

**Authors:** Reid Masterson, Travis Pebror, Andrew Gauger, Adam William Schmitz, Sabah David Butty

**Affiliations:** Department of Radiology and Imaging Sciences, Indiana University School of Medicine, 550 University Blvd, Room 0641, Indianapolis, IN 46202 USA

**Keywords:** Octogenarian, Advanced age, Pulmonary embolism, Thrombectomy, Reperfusion therapy

## Abstract

**Purpose:**

To evaluate outcomes in patients aged ≥ 80 years following large-bore aspiration thrombectomy (LBAT) for the treatment of pulmonary embolism (PE).

**Materials and methods:**

All patients ≥ 80 years of age with PE treated via LBAT at a single center were analyzed from September 2019 – August 2024. This included the octogenarian subgroup from a recently published retrospective analysis assessing all PE patients treated with LBAT at our center between September 2019 and January 2023. The following outcomes were evaluated: technical success, change in several hemodynamic measures including pulmonary artery pressure (PAP) and right ventricle to left ventricle ratio (RV to LV ratio), length of hospital and intensive-care-unit (ICU) stay, procedure-related complications, and 7- and 30-day mortality.

**Results:**

Forty-eight patients aged ≥ 80 years underwent LBAT procedures for PE. Technical success was achieved in 46 cases (95.8%). The mean reduction in mean PAP was 3.6 mmHg. The mean reduction in RV to LV ratio was -0.42. The mean length of postprocedural hospital and ICU stays were 5.7 ± 3.6 days and 1.0 ± 1.6 days, respectively. There were 2 procedural complications, 1 pulmonary vascular injury involving a pulmonary artery pseudoaneurysm and 1 decompensation involving hypotension requiring vasopressor support. There were no major bleeding complications or cardiac injuries. All-cause mortality was 2.1% (*n* = 1) at 7 days and 6.3% (*n* = 3) at 30 days post procedure. PE-related mortality was 2.1% (*n* = 1) at 30 days.

**Conclusion:**

LBAT is a technically feasible procedure for the treatment of PE in octogenarian patients and has a favorable preliminary safety and mortality profile.

## Introduction

Pulmonary embolism (PE) incidence increases with age. Elderly patients, particularly those > 80 years of age, have a higher mortality rate following PE than younger patients [1]. In octogenarians with acute PE, in-hospital and 30-day mortality has been estimated at 10% and 19%, respectively [1, 2]. Patients > 75 years are at higher risk of bleeding complications with anticoagulant and thrombolytic agents than younger patients [3, 4].

In the Pulmonary Embolism Thrombolysis (PEITHO) study, the rate of major extracranial bleeding in patients > 75 years was 11.1% [4]. Advanced age is generally regarded as a relative contraindication for systemic thrombolysis [5, 6].

Prior studies have suggested that catheter-directed therapies could prove safer for elderly PE patients who require reperfusion therapy [7–9]. To our knowledge, no study has evaluated the use of large-bore aspiration thrombectomy (LBAT) in this demographic. Here we describe the outcomes of patients aged ≥ 80 years who underwent LBAT for the treatment of PE at a large university medical center.

## Materials and methods

All patients ≥ 80 years of age with PE treated via LBAT using the FlowTriever System (Inari Medical, Irvine, CA) at our center between September 2019 and August 2024 were included. No exclusionary criteria were applied. The population included an octogenarian subgroup from a previously conducted retrospective analysis of all 286 PE patients treated with LBAT between September 2019 and January 2023 [10].

Outcomes include: 1. technical success, defined per the Society of Interventional Radiology (SIR) reporting standards as the ability to deliver, operate, and remove the catheter from the pulmonary artery (PA) [11]; 2. change in hemodynamic outcomes including heart rate, hemoglobin, RV to LV ratio, echocardiography evaluation of RV function, systemic arterial pressure, pulmonary arterial pressure (PAP), and supplemental oxygen requirement; 3. hospital and intensive-care-unit (ICU) stay; 4. procedure-related complications including major bleeding (defined as intracranial, intraocular, or retroperitoneal hemorrhage or any hemorrhage requiring transfusion and/or resulting in a hemoglobin decrease ≥ 5 g/dL [11]), pulmonary vascular injury, cardiac injury, or procedure-related clinical decompensation (defined as an acute physiological worsening of clinical status that posed an immediate increased risk of serious harm or death [12]); and 5. all-cause mortality at 7 and 30 days post-procedure. RV to LV ratio was measured on axial format computed tomography (CT). Echocardiography findings of impaired RV systolic function, RV dilation, or elevated RV systolic pressure were reported as described by the interpreting cardiologist.

The statistical analysis was descriptive in nature. Categorical values are reported as counts (%) and continuous variables are reported as mean ± standard deviation.

This research activity was granted exemption from full Institutional Review Board review and waived informed consent by a qualified staff member of the Human Research Protection Program of the affiliated university in accordance with 45 CFR 164.512(i)(2)(ii).

## Results

### Patient characteristics

A total of 48 patients aged ≥ 80 years underwent LBAT for PE. Baseline characteristics are shown in Table 1. The mean age was 83.8 years. All patients had elevated cardiac biomarkers (troponin-I and/or brain natriuretic peptide) and underwent diagnostic imaging with CT. Most patients had CT evidence of right ventricular dilatation (*n* = 41; 85.4%) with mean RV to LV ratio of 1.4 ± 0.4 and were classified as intermediate-high-risk PE (*n* = 38; 79.2%) per European Society of Cardiology guidelines [3]. Two patients (4.2%) had high-risk PE, each presented with a systolic blood pressure < 90 mmHg persisting for > 15 min.

**Table 1:** Baseline characteristics

Demographics	All octogenarians *N* = 48
Age, years	83.8 ± 3.8
Male sex	23 (47.9)
Body mass index, kg/m^2^	29.4 ± 6.9
PE risk factors	
History of PE	8 (16.7)
History of DVT	8 (16.7)
Obesity	17 (35.4)
Malignancy	14 (29.2)
Surgery within prior 3 months	4 (8.3)
Immobility for ≥ 30 days	12 (25.0)
Concurrent DVT	31 (64.6)
Presenting symptoms	
Dyspnea	42 (87.5)
Increased oxygen requirement	27 (56.3)
Tachycardia	14 (29.2)
Chest pain	16 (33.3)
Syncope/presyncope	17 (35.4)
Symptom duration, days	3.1 ± 3.0
Diagnostics	
Right ventricular dilatation on CT	41 (85.4)
RV to LV Ratio	1.7 (0.4)^a^
Elevated troponin-I or BNP	48 (100.0)
Elevated troponin-I	45 (93.8)
Elevated BNP	38 (79.2)
ESC risk stratification	
High-risk PE	2 (4.2)
Intermediate-high-risk PE	38 (79.2)
Intermediate-low-risk PE	8 (16.7)

Values are reported as mean ± SD or n (%)

*BNP* brain natriuretic peptide, *CT* computed tomography, *DVT* deep vein thrombosis, *ESC* European Society of Cardiology, *LV* left ventricle, *PE* pulmonary embolism, *RV* right ventricle

^a^Data missing for *n* = 2

### Procedure and hospital stay

All procedural characteristics and patient outcomes are shown in Table 2. Technical success was achieved in 46 cases (95.8%). The 2 unsuccessful procedures were performed in an 82-year-old intermediate-high-risk patient with chronic thrombus and an 80-year-old intermediate-low-risk patient who developed a PA pseudoaneurysm which was managed conservatively [10]. The mean procedure time from preprocedural time-out to access site closure was 85.9 ± 29.9 min. The mean fluoroscopy time was 22.2 ± 9.6 min. No patients received thrombolytics before, during, or after the procedure.

**Table 2:** Procedural characteristics and outcomes

	All octogenarians *N* = 48
**Procedural Characteristics**	
Procedural time, minutes	85.9 ± 29.9
Fluoroscopy time, minutes	22.2 ± 9.6
Technical success	46 (95.8)
**Hemodynamic Outcomes**	
Change in heart rate, beats/min	-5.0 ± 28.4
Change in hemoglobin, g/dL	-1.4 ± 1.4^a^
*RV to LV Ratio Change on CT*	
CT within 60 days of LBAT	10 (20.8)
Change in RV to LV Ratio	-0.42 (0.19)
*RV Evaluation by Echocardiogram*	
*Echocardiogram within 60 of LBAT*	20 (46.5)
*Impaired RV Systolic Function*	6 (30.0)
*RV Dilation*	4 (20.0)
*Elevated RV systolic pressure*	3 (27.3)^b^
*Pulmonary Artery Pressure Change, mmHg*	
Systolic PAP	-8.3 ± 9.7^c^
Diastolic PAP	-1.2 ± 4.1^c^
Mean PAP	-3.6 ± 4.8^c^
*Systemic Blood Pressure Change, mmHg*	
Systolic SAP	0.8 ± 20.6
Diastolic SAP	0.4 ± 12.7
Mean SAP	0.6 ± 12.9
*Supplemental Oxygen Requirement*	
Resolved following LBAT	11 (22.9)
Newly Required following LBAT	11 (22.9)
**Length of Stay**	
Postprocedural ICU stay, days	1.0 ± 1.6
No postprocedural ICU stay	31 (64.6)
Postprocedural ICU stay in patients with stay > 0, days	2.8 ± 1.5
Postprocedural hospital stay	5.7 ± 3.6
**Morbidity & Mortality**	
Major bleeding	0 (0.0)
Pulmonary vascular injury	1 (2.1)
Cardiac injury	0 (0.0)
Procedural decompensation	1 (2.1)
All-cause mortality at 7 days	1 (2.1)
All-cause mortality at 30 days	3 (6.3)
PE-related mortality at 30 days	1 (2.1)

Values are reported as mean ± SD or n (%)

*CT* computed tomography, *ICU* intensive care unit, *PAP* pulmonary artery pressure, *LV* left ventricle, *PE* pulmonary embolism, *RV* right ventricle

^a^Data missing for *n* = 1

^b^Data missing for *n* = 9

^c^Data missing for *n* = 4

Paired PAP measurements from pre- and post-thrombectomy were available for 44 patients (91.7%). Systolic PA pressure decreased from 44.7 ± 10.6 mmHg to 36.4 ± 11.0 mmHg; a mean reduction of 8.3 mmHg. Mean PA pressure decreased from 25.6 ± 6.9 mmHg to 22.0 ± 6.1 mmHg; a mean reduction of 3.6 mmHg. There were 10 patients (20.8) who underwent CT within 60 days following LBAT with mean decrease in RV to LV ratio of 0.42 ± 0.19. There were 20 patients who underwent echocardiogram within 60 days following LBAT. Of these patients there were 6 (30%) with impaired RV systolic function, 4 (20%) with RV dilation and 3 (15%) with elevated RV systolic function.

The average post-procedural ICU and hospital stays were 1.0 ± 1.6 days and 5.7 ± 3.6 days, respectively. Thirty-one patients (64.6%) had no post-procedure ICU stay. Among the 17 patients admitted to the ICU following the procedure, the mean length of ICU stay was 2.8 ± 1.5 days.

### Safety and mortality

There were 2 procedural complications (4.1%), one pulmonary vascular injury (previously described case of PA pseudoaneurysm [10]) and one intraprocedural decompensation involving hypotension treated with vasopressor. There were no major bleeds or cardiac injuries.

The 7-day all-cause mortality rate was 2.1% (*n* = 1). This death was considered PE-related and occurred in the patient for whom LBAT was not successful due to chronic thrombus. The patient died of progressive cardiogenic shock on post procedure day 1.

The 30-day all-cause mortality rate was 6.3% (*n* = 3). The 2 additional deaths between 7 and 30 days were not related to PE. These patients died due to cardiac arrest in the setting of palliative care and malignancy progression at post-procedure days 13 and 14, respectively.

## Discussion

These results suggest LBAT is a safe and effective treatment option for PE patients ≥ 80 years. This study is limited by its retrospective nature and sample size. However, these findings are important because despite experiencing higher PE incidence and mortality rates, octogenarians are often underrepresented in clinical studies.

Data from the National Inpatient Sample indicate patients ≥ 80 years comprise 18.4% of patients with an index admission for PE [1]. At our institution, patients aged ≥ 80 years represented 10.1% of all patients who underwent LBAT for PE over a > 3-year period (September 2019–January 2023) [10]. During this period, outcomes were similar for the octogenarian subgroup (*n* = 29) vs the full cohort (*n* = 286) including rate of procedural-related complications (3.4 vs 2.8%), proportion without postprocedural ICU admission (48.3 vs 49.3%), mean length of postprocedural hospital stay (6.2 vs 7.3 days), and 30-day PE-related mortality (3.4 vs. 3.5%) [10].

The 6.3% rate of 30-day all-cause mortality in this analysis is similar to prior studies of octogenarians [1, 2, 13]. Although reperfusion with systemic thrombolysis has been shown to reduce in-hospital mortality among octogenarians, it was associated with more overall bleeding complications and a 7% rate of major bleeding [13]. An analysis of patients ≥ 65 years with PE reported reduced major bleeding with catheter-based therapies compared with systemic thrombolysis and surgical embolectomy [7]. Al-Terki et al. reported no major bleeding with ultrasound-accelerated thrombolysis among 19 octogenarians with intermediate-high-risk PE [8]. Harrison et al. reported 1 major bleeding event with catheter-directed thrombolysis among 18 patients ≥ 65 years of age with intermediate-high-risk PE [9]. Consistent with prior studies, there were no major bleeding events in this analysis.

## Conclusion

In octogenarian patients, LBAT for the treatment of PE is a technically feasible procedure with a favorable preliminary safety and mortality profile through 30 days.

## Data Availability

The datasets generated and/or analyzed during the current study are not publicly available due to reasons of sensitivity and are available from the corresponding author on reasonable request. Data are located in controlled access data storage at Indiana University.
